# Mitochondrial-related genes PDK2, CHDH, and ALDH5A1 served as a diagnostic signature and correlated with immune cell infiltration in ulcerative colitis

**DOI:** 10.18632/aging.205561

**Published:** 2024-02-19

**Authors:** Qian Yang, Peng Zhang, Lu Han, Pengshuang Shi, Zhifang Zhao, Dejun Cui, Kunqiao Hong

**Affiliations:** 1 Department of Gastroenterology, Guizhou Provincial People’s Hospital, Medical College of Guizhou University, Guiyang, Guizhou, China; 2 Department of Urology, Guizhou Provincial People’s Hospital, Guiyang, Guizhou, China; 3 Department of Gastroenterology, Renmin Hospital of Wuhan University, Wuhan, Hubei, China

**Keywords:** ulcerative colitis, mitochondria, immune infiltration, pyruvate dehydrogenase kinase 2, aldehyde dehydrogenase 5 family member A1, choline dehydrogenase

## Abstract

We conducted an investigation to determine the potential of mitochondrial-related genes as diagnostic biomarkers in ulcerative colitis (UC), while also examining their association with immune cell infiltration. To achieve this, we acquired four datasets pertaining to UC, which included gene expression arrays and clinical data, from the GEO database. Subsequently, we selected three signature genes (PDK2, CHDH, and ALDH5A1) to construct a diagnostic model for UC. The nomogram and ROC curves exhibited exceptional diagnostic efficacy. Following this, quantitative real-time polymerase chain reaction and western blotting assays validated the decreased mRNA and protein expression of PDK2, CHDH, and ALDH5A1 in the model of UC cells and dextran sulfate sodium salt (DSS)-induced mice colitis tissues, aligning with the findings in the risk model. This investigation suggested a negative correlation between the expression of ALDH5A1, CHDH, and PDK2 and the infiltration of M1 macrophages. Then, immunofluorescence analysis confirmed the augmented expression of CD86 in the tissue of mice subjected to DSS, while a diminished expression of ALDH5A1, CHDH, and PDK2 was observed. Consequently, it can be inferred that targeting mitochondria-associated genes, namely PDK2, CHDH, and ALDH5A1, holds potential as a viable strategy for prognostic prediction and the implementation of immune therapy for UC.

## INTRODUCTION

Ulcerative colitis (UC) is a prevalent inflammatory bowel disease (IBD) that is distinguished by a multifaceted pathophysiological process, encompassing an aggressive immune response and the loss of the intestinal epithelium [1]. In recent years, UC has exhibited a growing incidence in all countries, thereby increasing its prevalence and economic and social burden [2]. The pathogenesis of UC is influenced by immunological, genetic, and environmental factors, which culminate in the persistent and inappropriate activation of the mucosal immune system [3, 4].

Extensive research has been conducted on the involvement of mitochondria in the progression of UC, with particular emphasis on the identification of mitochondrial genes that hold promise as potential targets for UC treatment [5]. Mitochondria are intricate organelles that play critical roles in bioenergetics, cellular homeostasis, and innate immunity, and their dysfunction has been implicated in various pathological conditions, including cardiovascular diseases [6], neurological disorders [7], and cancer [8]. Moreover, a recent investigation has revealed that the manifestation and advancement of UC are substantially influenced by mitochondrial dysfunction, primarily through its impact on ATP levels, mitochondrial reactive oxygen species (mtROS), mitochondrial damage-associated molecular patterns (DAMPs), and immune function [9].

The investigation of potential biomarkers and regulatory mechanisms for early diagnosis and therapeutic targets of UC holds significant scientific and practical importance. Despite the limited attention given to the differential analysis and prognosis model of mitochondria-related coding genes in recent years, the utilization of bioinformatics can facilitate the identification of potential diagnostic biomarkers, therapeutic targets, and pathogenesis of UC in the mitochondria. This approach can aid in predicting the diagnosis and prognosis of UC and the development of networks. In this investigation, we acquired gene expression data and corresponding clinical information from GEO datasets. We utilized the WGCNA package, differentially expressed analysis, LASSO logistic regression, and RandomForest assays to identify the three most significant feature genes. Subsequently, we employed nomograms, receiver operating characteristic (ROC) curves, and immune infiltration to evaluate the diagnostic and practical utility of the signature genes in UC. Ultimately, the verification of the expression of signature genes (ALDH5A1, CHDH, and PDK2) and their co-expression with M1 macrophages (CD86) was accomplished through the construction of inflammatory cell and colitis models. The objective of this research was to identify mitochondrial genes that could serve as diagnostic and prognostic biomarkers for UC, as well as molecular therapeutic targets based on the hypothesis of mitochondrial dysfunction.

## RESULTS

### Weighted gene co-expression network construction

**Figure 1:**
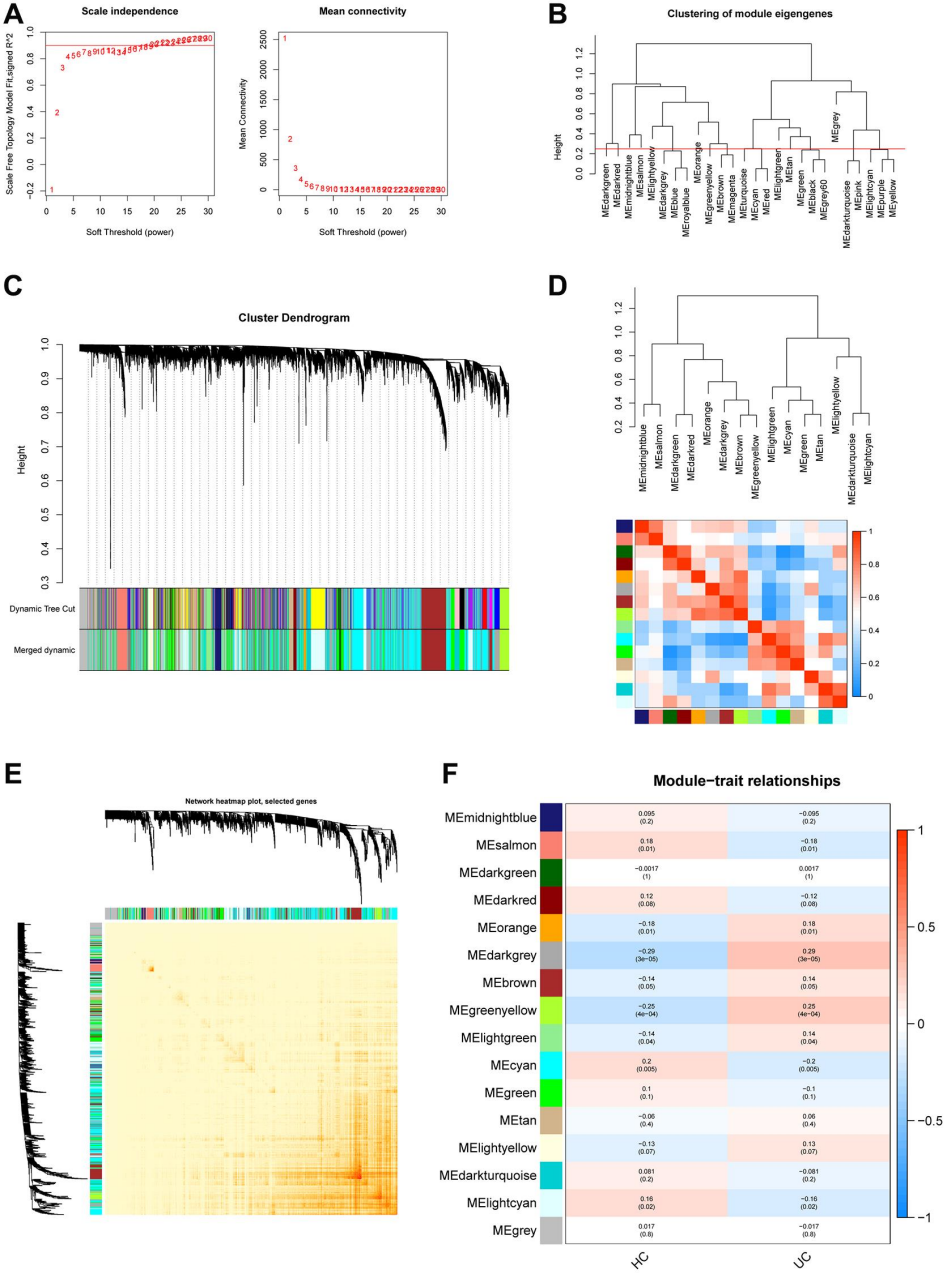
**Construction of WGCNA co–expression network in training set.** (**A**) The investigation involved the utilization of scale-free fit index and mean connectivity analysis across various soft-thresholding powers. (**B**) To identify and establish connections between related modules, the clustered dendrograms were truncated at 0.25 heights. (**C**) The WGCNA modules were assigned and represented by a colored horizontal bar in the cluster dendrograms. (**D**) The module feature genes were analyzed for collinearity and presented in a heatmap, where red indicates high correlation and blue indicates low correlation. (**E**) The topological overlap matrix (TOM) for each module was visualized in a WGCNA network heatmap. (**F**) Module-trait correlations. Positive correlations are represented by red and negative correlations by blue.

**Figure 2:**
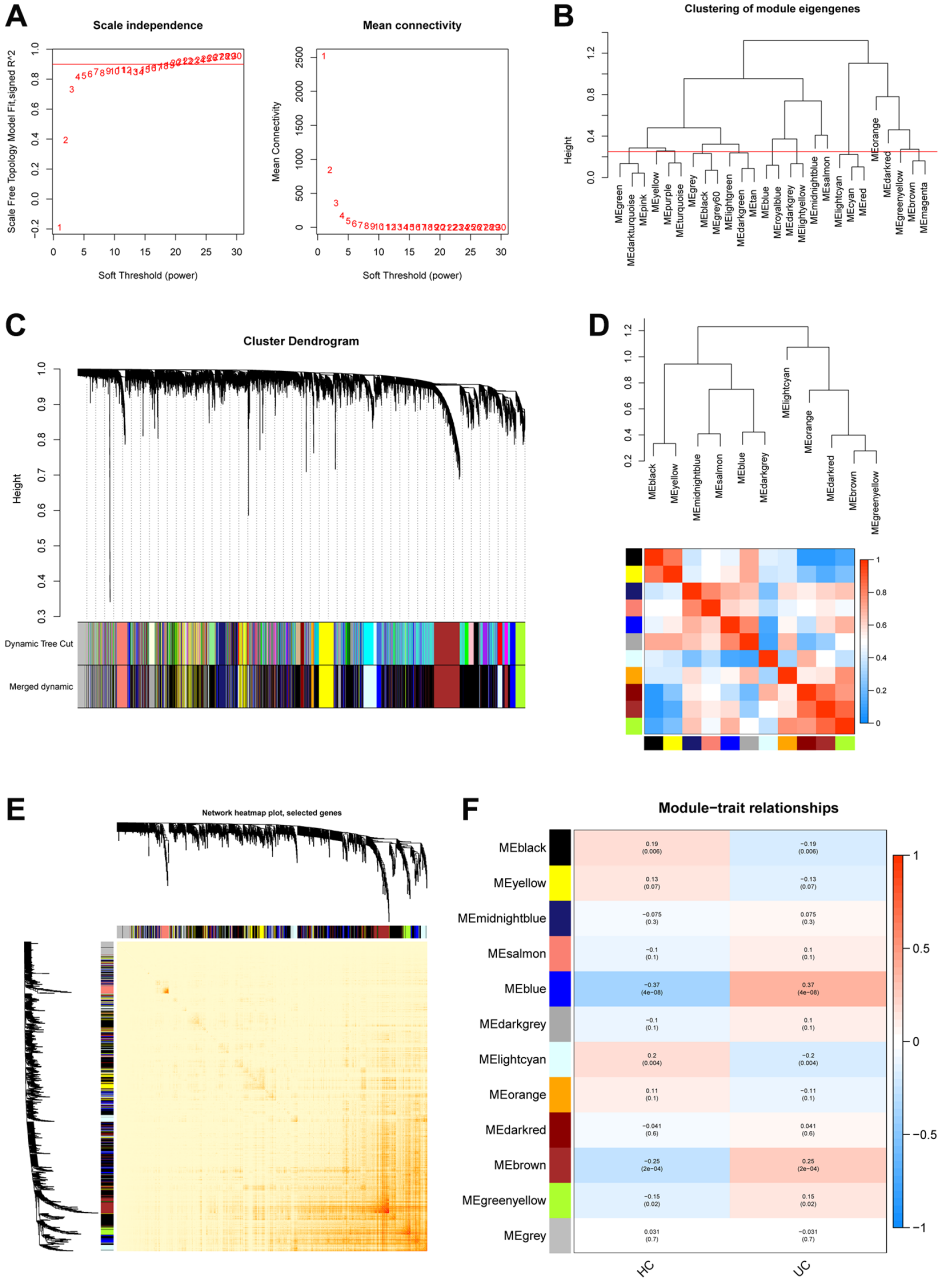
**Construction of WGCNA co–expression network in validation set.** (**A**) Scale-free fit index and mean connectivity analysis for various soft-thresholding powers. (**B**) Clustered dendrograms were cut at 0.25 heights to recognize and connect related modules. (**C**) Cluster dendrograms and module assignment for WGCNA modules. The colored horizontal bar represents the modules. (**D**) Collinear heat map of module feature genes. The color red represents a high correlation, whereas the color blue suggests the opposite results. (**E**) Visualization of the WGCNA network heatmap. The heatmap shows the topological overlap matrix (TOM) for each module. (**F**) Module-trait correlations. Positive correlations are represented by red and negative correlations by blue.

The GSE179285 and GSE107499 datasets were used as the training group, with 58 normal samples and 191 UC samples; and the GSE92415 and GSE48634 datasets were set as the testing group, with 47 normal samples and 111 UC samples. The 871 Mito-RGs isolated in the 302 UC samples were subjected to WGCNA analysis. In the following investigation, the value of the soft thresholding power was determined to be 7, as a result of the scale independence reaching 0.9 and having a reasonably high-average connectedness (Figures 1A and 2A). According to the merging of the highly linked modules at a clustering height limit of 0.25 (Figures 1B and 2B), 26 modules were chosen for in-depth analysis. Modules that had been primed and combined were finally shown in a clustering dendrogram (Figures 1C and 2C). To determine the connection between gene co-expression groups, eigen gene connectivity was evaluated. 14 modules were found to be divided into two clusters after we categorized the eigen genes (Figures 1D and 2D). Next, we established the connections between the recognized modules, and the analysis’s findings showed that gene expression was mostly independent between modules (Figures 1E and 2E). We explored significant correlations between modules and clinical features. In this analysis, the module darkgrey was most closely associated with UC (Figures 1F and 2F). A total of 1231 MRGs were found in the training group and 1140 MRGs in the testing group using WGCNA.

### Screening genes for differential expression

**Figure 3:**
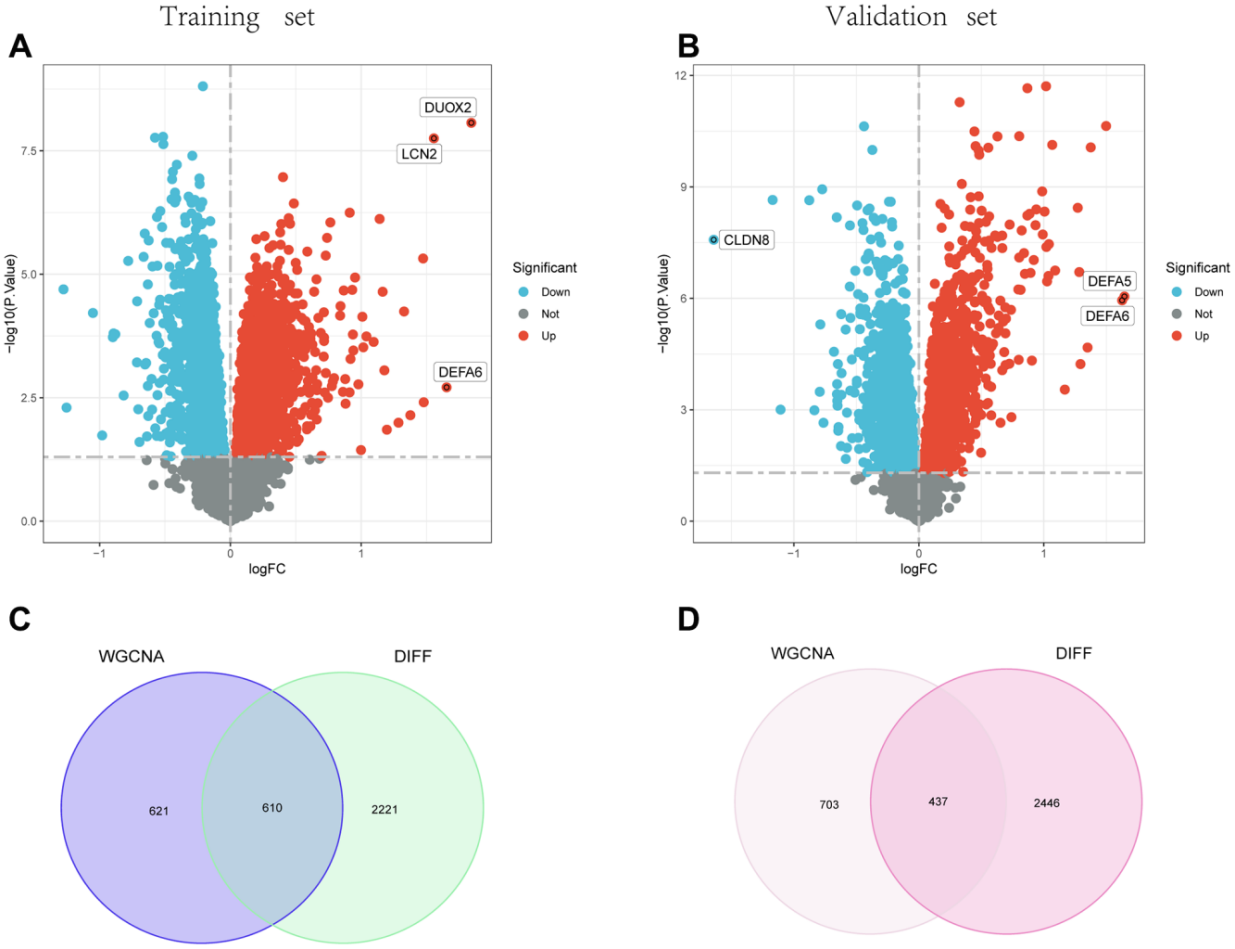
**Identification of DEGs.** (**A**) and (**B**) show the Volcano plot for DEGs between healthy controls and UC in the training and test groups, respectively. (**C**) and (**D**) Venn diagrams for intersections of DEGs and the WGCNA module in the training and test groups.

2831 genes in the training group and 2883 genes in the testing group were identified as differential expression genes (DEGs) under the criterion of *P*-adjustment 0.05 and |log_2_FC| >0.5. DEGs volcano plots are shown in Figure 3A, 3B, respectively, for the training group and the test group. After that, 610 intersecting genes were obtained in the training group (Figure 3C), and 437 intersecting genes were acquired in the testing group (Figure 3D) based on the intersections of the WGCNA analysis and the differential expression analysis.

### Hub genes identification and functional enrichment analysis

**Figure 4:**
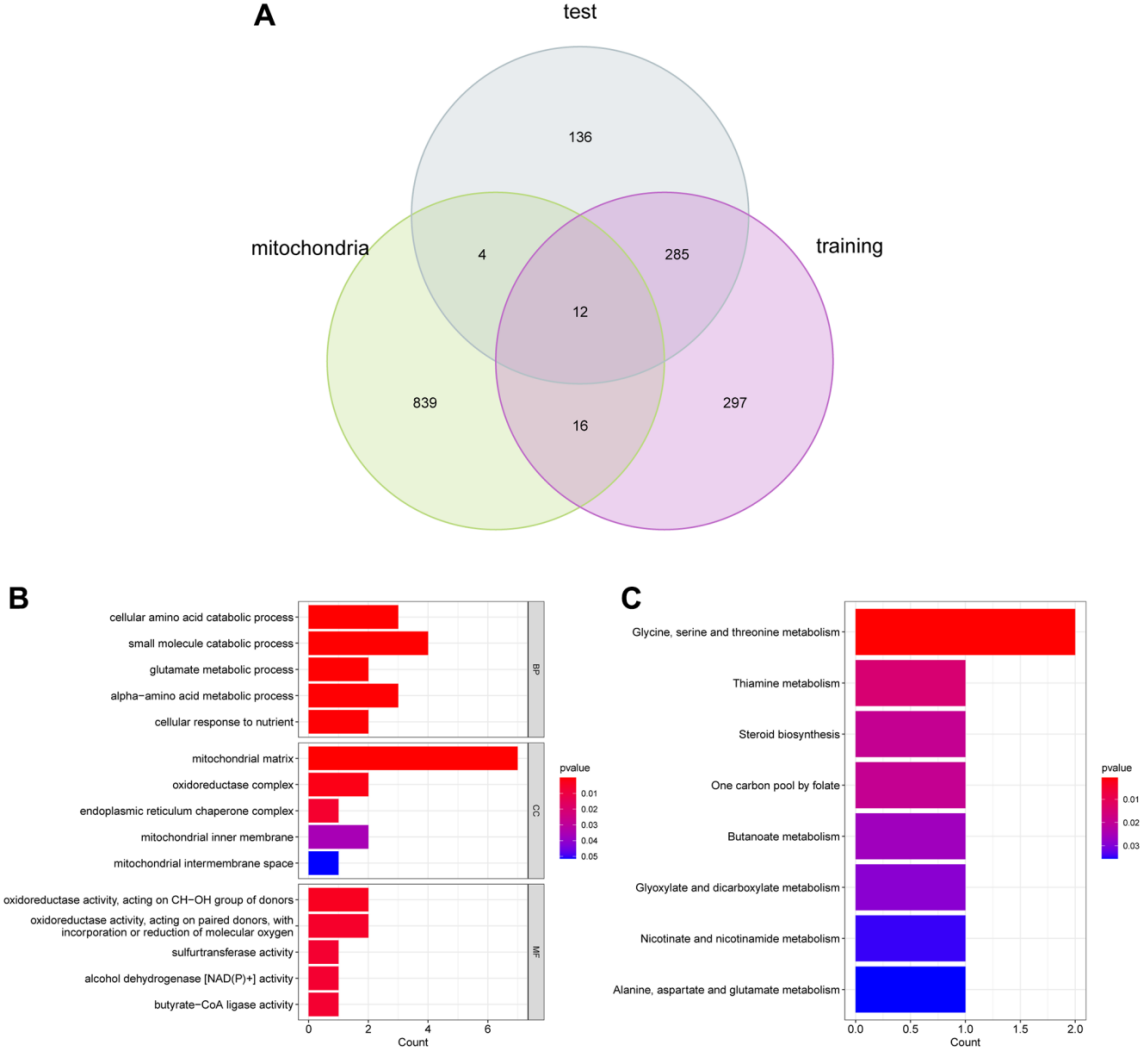
(**A**) Functional enrichment analysis. (**B**) and (**C**) GO and KEGG analysis of DEGs.

Using Venn diagram, we detected 12 UC-Mito DEGs via overlapping MRGs and important DEGs (Figure 4A). To discover the biological roles played by the hub genes in the modules, we conducted a functional analysis. All of the 12 UC-Mito DEGs were shown to be functionally enriched, and the GO barplot showed 15 GO keywords according to *P* < 0.05 (Figure 4B). According to the data, the enrichment of the biological process (BP) was predominantly linked to the cellular amino acid catabolic process, small molecule catabolic process, and glutamate metabolic process. Oxidoreductase activity, acting on CH-OH group of donors, oxidoreductase activity, acting on paired donors, and sulfur transferase activity are all connected to enriched molecular function (MF). The enrichment of cellular components (CC) is connected to the mitochondrial matrix, oxidoreductase complex, and endoplasmic reticulum chaperone complex. In KEGG analysis, the signaling pathway of glycine metabolism, thiamine metabolism, and the steroid biosynthesis were related (Figure 4C).

### Identification and evaluation of signature genes

**Figure 5:**
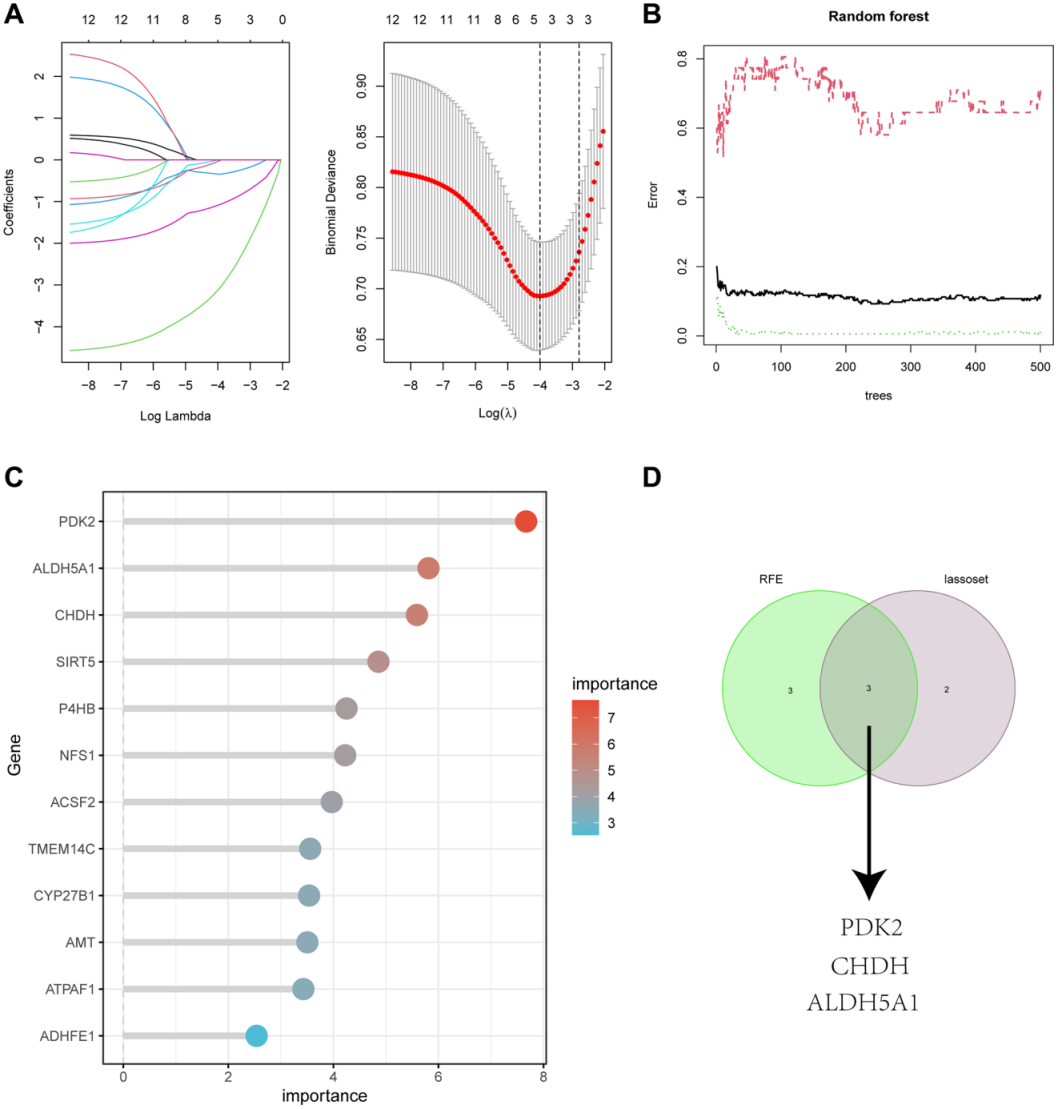
(**A**) Adjustment of feature selection in the minimum absolute shrinkage and selection operator model (lasso). (**B**) Random forest error rate versus the number of classification trees. (**C**) The top 12 relatively important genes. (**D**) Three algorithmic Venn diagram screening genes.

We used two machine algorithms to identify the most significant feature genes with predictive values: LASSO regression analysis to select 5 predicted genes from statistically significant univariate variables (Figure 5A) and RandomForest combined with feature selection to determine the relationship between error rate, number of classification trees (Figure 5B), and 6 genes with relative importance (Figure 5C). Using the intersection of the two methods described above, we produced a Venn diagram to identify three overlapping genes, PDK2, CHDH, and ALDH5A1. (Figure 5D).

**Figure 6:**
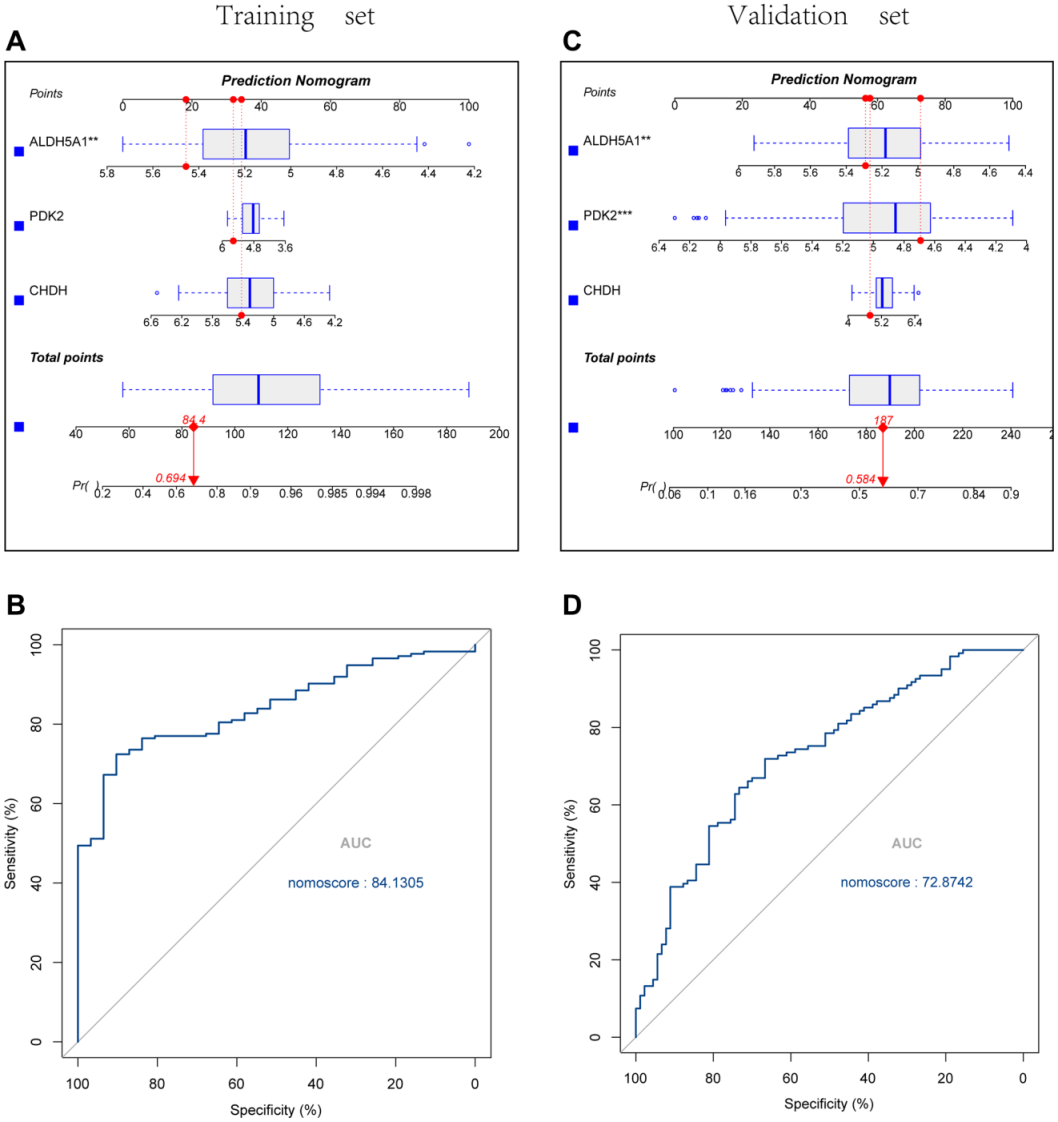
**Construction and validation of the UC diagnostic column line graph model.** (**A**, **B**) Column line graphs are utilized to predict the occurrence of UC in the training and validation cohorts. (**C**, **D**) ROC curves were created, with an area under the curve of 84.1305 in the training set and 72.8742 in the validation set.

Using the Rms package, we constructed UC diagnostic column line graph models for the signature genes (PDK2, CHDH, and ALDH5A1) (Figure 6A, 6C), and we evaluated the predictive value of these models using Receiver operating characteristic curves analysis. ROC curves were constructed with an area under the curve that was 84.1305 in the training set and 72.8742 in the validation set respectively (Figure 6B, 6D).

### Analysis of immune infiltration

**Figure 7:**
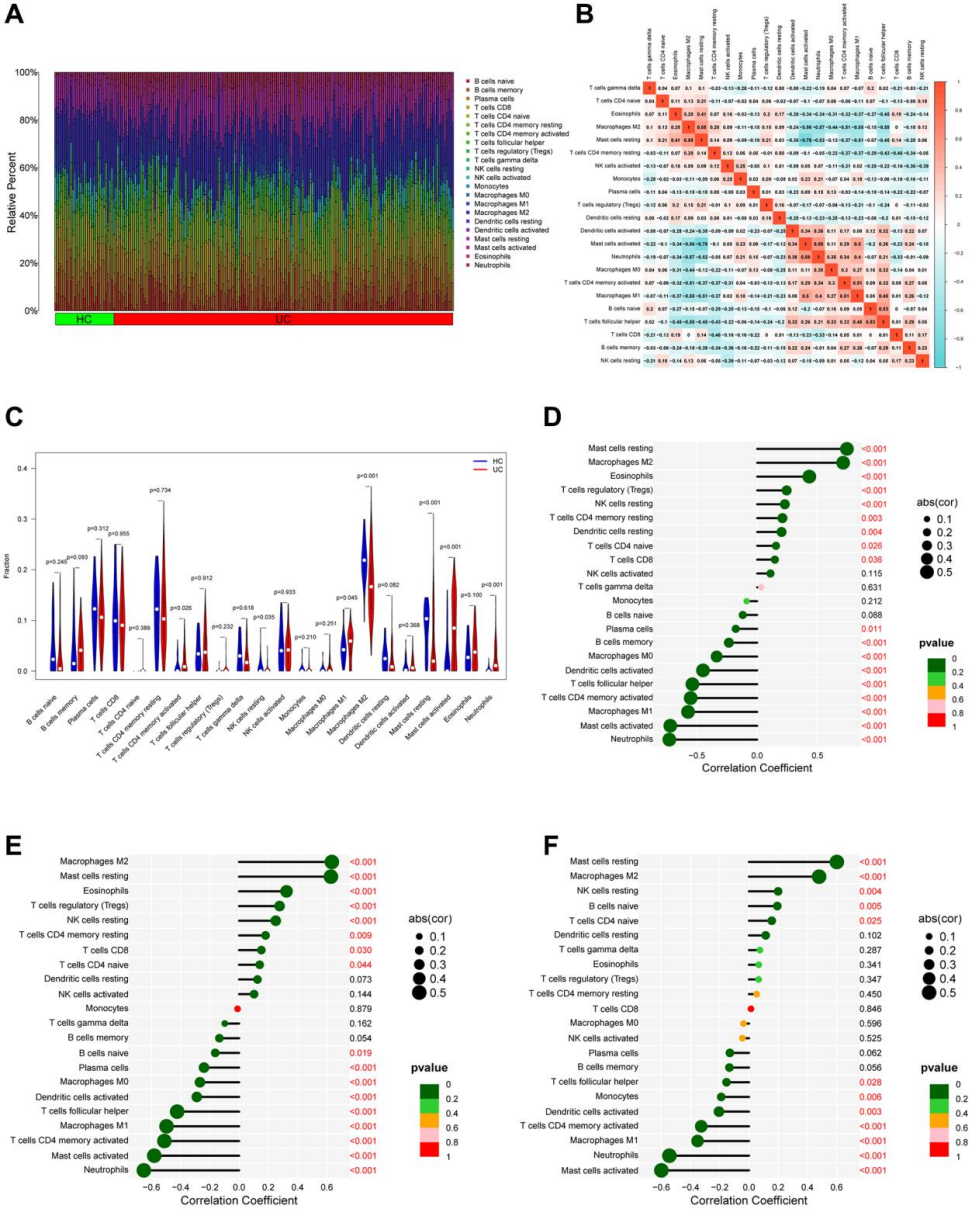
**Analysis of immune infiltration.** (**A**) Bar plot bar plot depicting the 22 subpopulations of immune cells in UC and healthy control (HC) samples. (**B**) Correlation heatmap of immune cells in UC samples. (**C**) Violin plot illustrating the varying proportions of immune cells between UC and normal control (NC). (**D**–**F**) Bubble plots demonstrate the relationship between immune cells and specific signature genes (PDK2, CHDH, and ALDH5A1).

Given the importance of the immune response to the development of UC, we aimed to describe the immunological infiltration landscape in UC. Using the CIBERSORT method, we compared UC samples with healthy control samples to determine whether or not there was a difference in the infiltration of 22 different types of immune cells (Figure 7A). Meanwhile, Figure 7B depicts the relevant heatmap analysis of immune cells. Neutrophils were positively correlated with activated mast cells (r = 0.58), whereas mast cells at rest were negatively associated with activated mast cells (r = −0.78). When compared to the healthy control, UC patients had higher levels of T cells CD4 memory activated, M1 macrophages, mast cells activated, neutrophils plasma cells, according to violin plots (Figure 7C) of the immune landscape created from the CIBERSORT algorithm (*P* < 0.05). To gain additional insight into the function of PDK2, CHDH, and ALDH5A1 in UC immune response, the correlation between the expression of signature genes and various degrees of immune cell infiltration was analyzed using the corrplot software. The results showed that PDK2 linked closely with Mast cells at rest (correlation = 0.74, *P* < 0.001) and negatively with M1 macrophages (correlation = −0.54, *P* < 0.001), as shown in Figure 7D. Figure 7E demonstrates that CHDH showed a positive association with M2 macrophages (correlation = 0.63, *P* < 0.001) and a negative correlation with M1 macrophages (correlation = −0.51, *P* < 0.001). As shown in Figure 7F, ALDH5A1 was positively correlated with Mast cells at rest (correlation = 0.74, *P* < 0.001) and negatively correlated with M1 macrophages (correlation = −0.39, *P* < 0.001). Furthermore, we employed xCELL algorithm to validate the correlation between immune cells and PDK2, CHDH, and ALDH5A1. The result was demonstrated in Supplementary Figure 1. These data also revealed that signature genes were closely associated with immune cells in UC.

### Validation of signature gene expression

**Figure 8:**
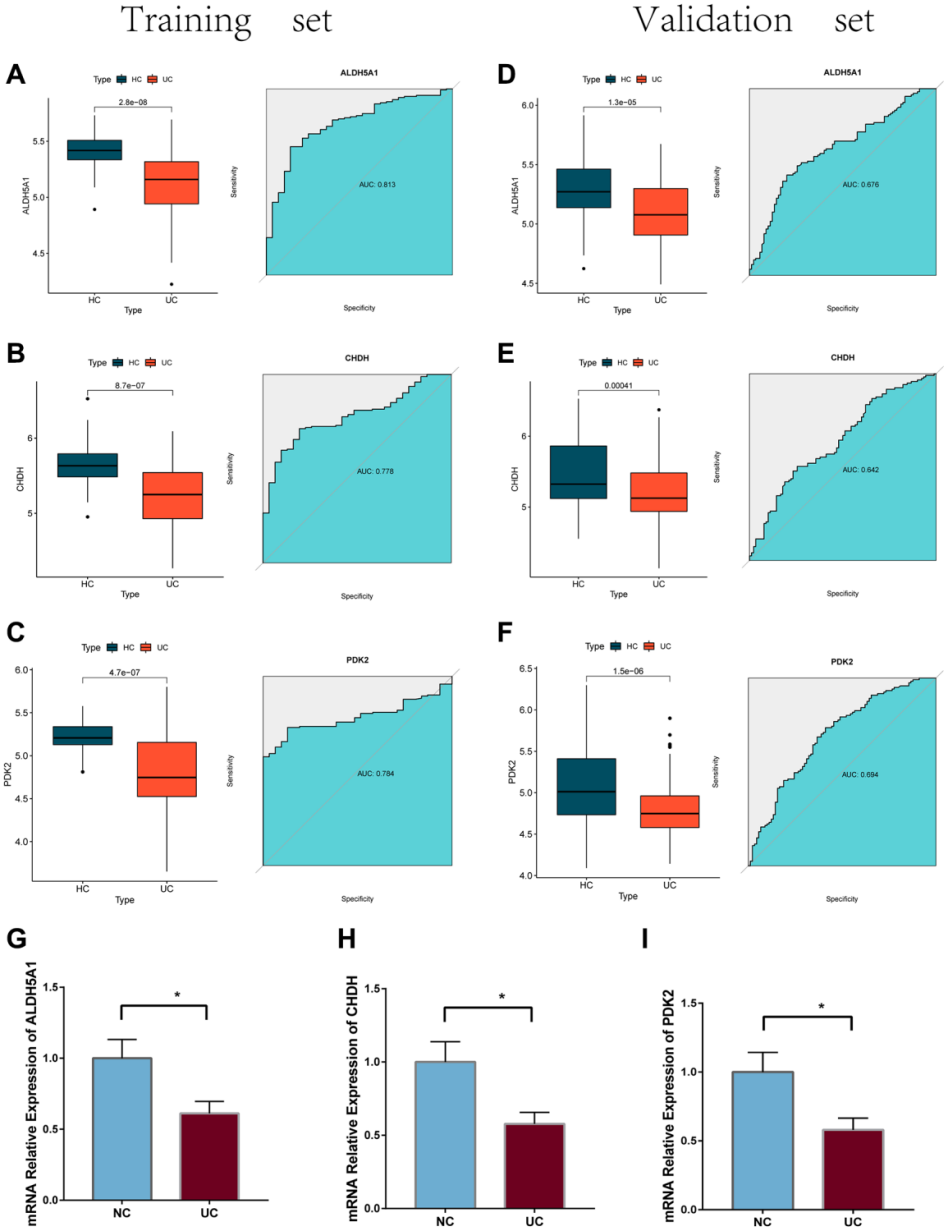
(**A**–**C**) The mRNA expression levels of the feature genes in the training cohort, as well as their ROC curves. (**D**–**F**) expression levels and ROC curves for the feature genes present in the training cohort. (**G**–**I**) the mRNA expression of three feature genes were validated using the LPS-induced NCM460 cells.

We evaluated the expression of these three genes in both the training and validation cohorts of UC patients and UC cells model (Figure 8). The ROC analysis was carried out so that we could further validate the diagnostic value of PDK2, CHDH, and ALDH5A1. It was discovered that PDK2 (AUC: 0.784), CHDH (AUC: 0.778), and ALDH5A1 (AUC: 0.813) all had better diagnostic values. The following findings were also confirmed by the validation datasets: PDK2 (AUC: 0.676), CHDH (AUC: 0.642), and ALDH5A1 (AUC: 0.694). (Figure 8A–8F).

**Figure 9:**
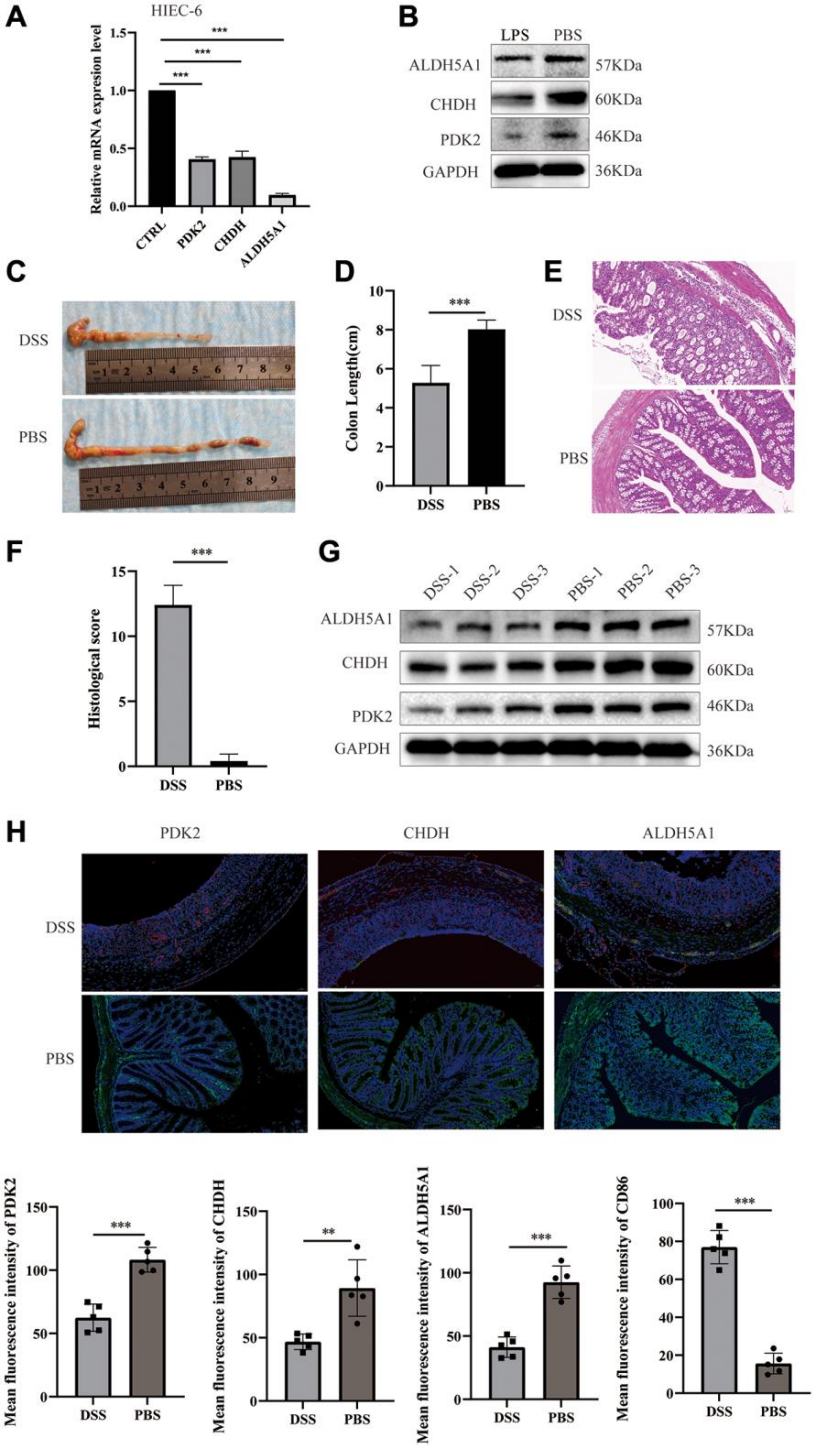
**Expression of PDK2, CHDH, and ALDH5A1 in LPS-induced HIEC-6 cells and DSS-induced colitis mice.** The relative mRNA (**A**) and protein (**B**) expression levels of PDK2, CHDH, and ALDH5A1 were conducted using the LPS-induced HIEC-6 cells. (**C**) Photographs were utilized to assess the variations in colon lengths among the groups. (**D**) The length of the colon in different groups. (**E**, **F**) Hematoxylin and eosin staining images and colonic histological scores of colon cross-sections in mice (*n* = 5). Scale bar = 50 μm. (**G**) Expression of PDK2, CHDH, and ALDH5A1 in the colon tissues of mice was detected using Western blotting. (**H**) Double immunofluorescence staining of PDK2 (labeled in green), CHDH (labeled in green), and ALDH5A1 (labeled in green) with M1 macrophages (CD86, labeled in red) at DSS induced mice. The nuclei were stained by DAPI (blue). *n* = 5 per group. Scale bar = 50 μm. Values are expressed as the mean ± sd (*n* = 5). ^*^P < 0.05, ^**^P < 0.01, ^***^P < 0.001. Abbreviation: DSS: dextran sodium sulfate.

Furthermore, the quantitative real-time PCR technique was employed to ascertain the downregulation of PDK2, CHDH, and ALDH5A1 expression levels in LPS-induced NCM 460 cells (Figure 8G–8I) and HIEC-6 cells (Figure 9A). The western-blotting method corroborated the decreased expression of ALDH5A1, PDK2, and CHDH in LPS-induced HIEC-6 cells (Figure 9B). To substantiate the involvement of ALDH5A1, PDK2, and CHDH *in vivo*, colitis was induced using a 3% DSS solution, resulting in a shorter colon length in the colitis group compared to the control (PBS) group (Figure 9C, 9D). Histological analysis revealed conspicuous infiltration of inflammatory cells in the intestinal mucosa and extensive epithelial erosion in DSS-induced mice (Figure 9E, 9F). Additionally, we assessed the protein expression levels of ALDH5A1, PDK2, and CHDH in the colon tissues of DSS-treated mice. Western blotting analysis demonstrated a reduction in the protein expression levels of ALDH5A1, PDK2, and CHDH in the colitis lesions of mice administered with DSS (Figure 9G).

Subsequently, immunofluorescence analysis was conducted to evaluate the association between immune cell infiltration and the expression of PDK2, CHDH, and ALDH5A1. The expression of CD86 (antigen-presenting surface molecules of M1 macrophage) was examined in relation to PDK2, CHDH, and ALDH5A1 expression. We found that CD86 was not detectable in control specimens. And the results also demonstrated that CD86 exhibited higher expression in mouse colitis lesions, whereas PDK2, CHDH, and ALDH5A1 displayed lower expression in these lesions (Figure 9H). Collectively, our findings indicate that the influence of PDK2, CHDH, and ALDH5A1 on the advancement of DSS-induced colitis in mice is potentially mediated through the regulation of macrophage infiltration. Nevertheless, the precise mechanism underlying the moderation of colitis remains to be elucidated.

## DISCUSSION

Ulcerative colitis (UC) is a multifaceted, chronic, immune-mediated intestinal inflammation disease [1]. Despite its increasing prevalence worldwide in recent decades, the etiology and pathogenesis of UC remain unknown. A comprehensive assessment, typically involving clinical symptoms, laboratory tests, and endoscopic examinations, is required for its diagnosis, which can be challenging [10]. Common symptoms of UC include diarrhea and blood in the stool. However, prolonged drug use may lead to significant adverse consequences [10]. Consequently, the exploration of innovative genetic biomarkers would provide a new outlook on the diagnosis and treatment approaches for UC.

The depletion of mucosa and intestinal stem cells is a result of mitochondrial dysfunction caused by the high energy demands of intestinal epithelial cells [11]. The production of reactive oxygen species (ROS) is significantly influenced by mitochondrial oxidative phosphorylation, which in turn affects gene regulation, DNA damage, ion transport, intermediate metabolism, and mitochondrial function. [12]. By enhancing mitochondrial function and reducing ROS and oxidative damage, UC can be mitigated [13, 14]. Mitochondrial DNA functions as a circulating damage-associated molecular pattern that is discharged from the active mucosa of UC, thereby contributing to the inflammatory process [15]. Additionally, Xu et al. suggest that dysfunctional mitochondria play a role in regulating immune cell homeostasis through pro-inflammatory cytokines, mitochondrial antigen presentation, and mitophagy. Consequently, addressing mitochondrial dysfunction presents a novel therapeutic approach for UC [16].

In this investigation, gene expression data were procured from the GEO database, while mitochondrial-related genes were acquired from MITOMAP. A total of 610 UC-related genes were scrutinized in the training set, and 437 genes were screened in the validation set, utilizing differential expression and WGCNA analysis, respectively. Through the intersection of mitochondrial genes, UC-related genes in the training and validation sets, we were able to identify 12 UC-Mito DEGs. Furthermore, performing LASSO logistic regression and RandomForest, pick out the 3 most important feature genes (PDK2, CHDH, and ALDH5A1). The nomogram showed the well diagnostic efficacy of the model constructed with these signature genes for UC. The calibration curve for the nomogram and ROC curves of the risk score showed that predictive value of the model is credible. Similarly, the ROC curves of the 3 signature genes showed that these genes played an important role in the pathogenesis of UC.

Pyruvate dehydrogenase kinase (PDK) inhibits pyruvate dehydrogenase (PDH) by phosphorylating Ser293 on the E1 subunit of PDH (PDHE1alpha), so restricting PDH from converting pyruvic acid to acetyl coenzyme A and accessing the TCA cycle in mitochondria [17]. Among the four pyruvate dehydrogenase kinase isoforms (PDK1, PDK2, PDK3, and PDK4), PDK2 has the widest distribution [18]. PDK2, also known as pyruvate dehydrogenase kinase 2, is an enzyme involved in the regulation of mitochondrial energy metabolism. Specifically, PDK2 phosphorylates and inactivates PDH, a key enzyme that converts pyruvate into acetyl-CoA to enter the citric acid cycle and generate ATP in the mitochondria [19]. Growing evidence suggests that PDK2 influences glucose metabolism which is consequently attributed to tumor development [20, 21]. Sachiko et al. found that high PDK2 expression was associated with a poor prognosis and that inhibiting PDK2 increased cisplatin sensitivity by activating the electron transport chain and increasing the generation of mitochondrial reactive oxygen species in Ovarian clear cell carcinoma [22]. Moreover, Li et al. found that VSIG4 activates the PI3K/Akt-STAT3 pathway, leading to PDK2 upregulation and subsequent phosphorylation of pyruvate dehydrogenase, which results in a reduction in pyruvate/acetyl-CoA conversion, mitochondrial reactive oxygen species secretion [23]. It is well known that the signal transducer and activator of transcription 3 plays an important role in regulating various physiological functions in UC [24, 25]. Additionally, Lower PDK2 protein levels were associated with chronic periodontitis [26], and PDK2 modulates metabolic and inflammatory processes in the hypothalamus [27]. When PDK2 is reduced, PDH becomes more active and promotes mitochondrial function by increasing the production of ATP. The depletion of PDK2 has the potential to cause mitochondrial dysfunction by disrupting the equilibrium between energy provision and consumption. This may culminate in a decline in ATP synthesis and an escalation in ROS generation, thereby impairing mitochondrial DNA and proteins, ultimately resulting in cellular demise and tissue impairment [28]. Consequently, it is imperative to conduct additional research on the involvement of PDK2 in UC and devise specific interventions to reinstate mitochondrial activity and mitigate the advancement of the disease.

Targeting mitochondrial metabolism and redox homeostasis is an attractive therapeutic strategy for improving drug sensitivity. Choline dehydrogenase (CHDH) as the mitochondrial enzyme is responsible for catalyzing the dehydrogenation of choline into betaine aldehyde and regulates mitophagy by recruiting SQSTM1 and LC3 to the mitochondria [29]. In recent years, studies have shown the importance of mitophagy within the intestinal epithelium during UC pathogenesis [16, 30, 31]. CHDH also primarily contributes to biological processes such as inflammatory response, vascular lesion, metabolic process and cell cycle [32]. Besides, CHDH was associated with an increased risk of breast cancer [33] and esophageal cancer [34]. A decrease in CHDH expression has been observed in UC, which is believed to be a contributing factor to the mitochondrial dysfunction observed in this disease. The mitochondrial dysfunction in UC has been associated with elevated levels of reactive oxygen species and compromised energy metabolism, both of which have the potential to cause tissue damage and inflammation [35]. Hence, the restoration of CHDH expression or activity presents a promising therapeutic approach for ameliorating mitochondrial function and mitigating inflammation in UC.

Aldehyde dehydrogenase 5 family member A1 (ALDH5A1), which encodes for Succinate semialdehyde dehydrogenase, is an enzyme that participates in mitochondrial glutamate metabolism, thereby regulating mitochondrial energy metabolism and contributing to the maintenance of mitochondrial integrity. Evidence suggests that the depletion of ALDH5A1 can result in mitochondrial dysfunction, including the impairment of γ-aminobutyric acid (GABA) metabolism [36]. Research has demonstrated that a decrease in ALDH5A1 may result in mitochondrial dysfunction, characterized by diminished ATP production, heightened oxidative stress, and impaired mitochondrial dynamics, ultimately culminating in cellular apoptosis and inflammatory responses [37]. Within the context of UC, the reduction of ALDH5A1 can exacerbate mitochondrial dysfunction and oxidative stress, thereby contributing to the advancement of the disease. Therefore, PDK2, CHDH, and ALDH5A1 may be involved in the etiology and progression of UC.

In order to confirm the expression of PDK2, CHDH, and ALDH5A1 in UC, both *in vitro* and *in vivo* experiments were conducted. The results demonstrated a downregulation of mRNA and protein expression levels of PDK2, CHDH, and ALDH5A1 in colitis cells and tissues, which aligns with our previous bioinformatic analysis. Given the critical role of the immune response in the pathogenesis of UC, we employed the CIBERSORT algorithm to assess the immune cell function in this disease. Our investigation revealed differential expression of seven immune cells in UC samples, suggesting their potential involvement in the initiation and progression of UC. Specifically, we observed significant differences in macrophages M1/M2, Mast cells resting, Mast cells activated, and Neutrophils. Notably, our immune cell profile of UC is consistent with prior research [38].

The macrophage subtypes, namely M1-like and M2-like macrophages, originate from monocytes and undergo dynamic transformations into various subtypes based on local conditions [39]. M1-like macrophages, stimulated by Th1 cytokines such as IFN-γ, play a crucial role in inflammation, pathogen inactivation, and tissue damage. These entities exhibit distinctive features, namely the secretion of pro-inflammatory cytokines such as IL-6, IL-1β and TNF-α, as well as the upregulation of antigen-presenting surface molecules including CD86, CD80, and CD83. This phenomenon elicits an augmented immune response through their interaction with T cells and other immune cells [40].

The results of our prior analysis indicate a negative correlation between M1 macrophages and the expression of PDK2, CHDH, and ALDH5A1. To further support this finding, we conducted immunofluorescence analysis to observe the co-localization of CD86 with PDK2, CHDH, and ALDH5A1. Our findings revealed that CD86 was not detectable in the control colon tissues, whereas its expression was increased in colitis tissues. Additionally, the expression levels of PDK2, CHDH, and ALDH5A1 were lower in colitis tissues, which aligns with the results obtained from the bioinformatic analysis. These observations indicate that the involvement of PDK2, CHDH, and ALDH5A1 in the modulation of the immune response may potentially contribute to the advancement of UC. Nevertheless, additional research is necessary to authenticate the importance of the immune response by means of integrated regulation of signature genes and immune cells in the context of UC.

In conclusion, the study established a correlation between these mitochondrial genes and immune cells. These findings were obtained through a comprehensive analysis of the GEO database, and the identified genes lack sufficient validation from existing studies. To further elucidate these findings, additional experimental validations using *in vivo* and *in vitro* approaches are imperative.

## MATERIALS AND METHODS

### Data processing and download

The gene expression array data and corresponding clinical datasets (GSE179285, GSE107499, GSE92415, and GSE48634) were retrieved from the Gene Expression Omnibus database. The training set for this study consisted of GSE179285 and GSE107499, while the validation set comprised GSE92415 and GSE48634. The former dataset included healthy controls (*n* = 58), ulcerative colitis patients with paired uninflamed colon (*n* = 48), and inflamed colon (*n* = 46), while the latter dataset included uninflamed colon (*n* = 22) and inflamed colon (*n* = 97). The GSE92415 dataset comprised healthy controls (*n* = 21) and ulcerative colitis patients (*n* = 87), and the GSE48634 dataset included healthy controls (*n* = 26) and ulcerative colitis patients (*n* = 24). Mitochondrial-related genes in the present study were downloaded from MSIGDB [3] (https://www.gsea-msigdb.org/gsea/msigdb/genesets.jsp?collection=H).

### Weighted gene co-expression network construction

The present study employed the R package “WGCNA” to extract expression profiles of both UC and healthy controls, thereby establishing a weighted gene co-expression network [41]. The evaluation of scale independence and mean connectivity was performed using the “pick soft threshold function.” Subsequently, modules were identified through hierarchical clustering and the dynamic tree cut function. The modules were then linked to disease features, and gene significance and module membership calculations were conducted. Further analysis was carried out on the matching module gene details. Finally, we displayed the eigen genes network.

### Identification of differentially expressed mitochondria-related genes

The MRGs expression profile was obtained from the transcriptome data that was downloaded, and a differential expression analysis was performed between healthy controls and UC patients using criteria of |Log fold change (FC)| >1 and false discovery rate (FDR) adjusted *P* < 0.05. The “ggplot” package was employed to generate a volcanic plot for visualization purposes.

### The acquisition of key genes

Initially, the intersecting genes were chosen from the Weighted Gene Co-expression Network Analysis (WGCNA) and differential expression analysis (diff) in the training set. Subsequently, the intersecting genes were selected from the WGCNA and diff in the validation set. Following this, the common genes between the aforementioned steps and the mitochondrial genes were identified and deemed as pivotal genes for further analysis.

### Functional enrichment analysis

Genes in interesting modules were taken out for further functional enrichment analysis. Essential genes’ biological properties were predicted and analyzed using a Gene Ontology analysis (GO) [42]. To find functional characteristics, KEGG pathway enrichment analysis was carried out [43]. The cut-off for significance was *P* < 0.05.

### Identification and evaluation of signature genes

The top gene list was produced using the least absolute shrinkage and selection operator (LASSO) analysis, which was used to exclude confusing genes and screen important genes for relationships between them. Additionally, we employed random forest for feature selection, which has been commonly used and can accurately evaluate the significance of each feature in the dataset, to pick out more meaningful key genes. With the use of LASSO logistic regression and RandomForest, the intersection was then used to pick out the study’s most important feature genes. Training set and validation set further confirmed the expression of important genes. The “Rms” program in R was used to produce nomograms, which were then used to determine the independent prognostic factors. The “Rms” program in R was used to produce nomograms, which were then used to determine the independent prognostic factors. To calculate the area under the curve (AUC) and assess the diagnostic value of the signature genes, we utilized the receiver operating characteristic (ROC) curves in the R package.

### Immune infiltration analysis

The CIBERSORT method was employed to investigate the proportion and composition of immune cells in UC and normal samples. Following that, immune cells correlation heatmap was constructed using the “corrplot” package. In order to show the dispersion of the Immune cells, the “vioplot” R package also was used. The Spearman rank correlation coefficient was calculated using the “corrplot” package to further investigate the link between immune cells and certain genes expression.

### UC cells model construction

NCM460 and HIEC-6 cell line were obtained from the Key Laboratory of Hubei Province for Digestive System Disease (Wuhan, Hubei, China) and cultured in RPMI-1640 medium (SH30809; HyClone, USA) and Cytiva HyClone™ DMEM/F12 (SH30023.01; HyClone, USA) containing 10% fetal bovine serum (FBS, sijiqing, Hangzhou, China) at 37°C in a humidified atmosphere of 5% CO_2_. NCM460 and HIEC-6 cells were seeded and then treated with LPS (1 μg/ml; Sigma) for 24 h to construct the UC cells model *in vitro*. After 24 h, LPS-induced NCM460 and HIEC-6 cells were harvested for further experiments.

### Animals and induction of colitis

Male C57BL/6 mice, aged 6 to 8 weeks and weighing 15–20 g, were procured from Hubei Biont Biological Technology Co., Ltd. (Wuhan, China). The mice were housed in a controlled environment with a 12-hour light/dark cycle and maintained at a standard temperature of 20 ± 2°C. Prior to the commencement of the experiment, the mice were acclimatized to these conditions for a minimum of 7 days. All animal procedures were approved by the Animal Ethics Committee of Guizhou Provincial People’s Hospital (Animal, 2022-039). From day 8 to day 14, the mice were administered sterile drinking water supplemented with 3% (w/v) dextran sulfate sodium salt (DSS, MP Biomedicals, Irvine, CA, USA) to induce experimental murine colitis. The control group received sterile drinking water. Following a 1-week period of 3% DSS treatment, mice were anesthetized and euthanized. The length of the colon was measured after isolation. A portion of the distal colon was then fixed with 4% paraformaldehyde (Biosharp) at room temperature for 24 hours to facilitate hematoxylin and eosin staining. The remaining sections were promptly frozen in liquid nitrogen for future assays. The histological scores were determined based on established scoring criteria.

### Quantitative real-time PCR

The total RNA of cells and tissues were extracted from Trizol (Cat#9109; TaKaRa, Japan), and the PrimeScript RT Master Mix kit was used to reverse it into cDNA (Cat#RR047A; Takara, Japan). All of the mRNA levels were determined by employing the SYBR Green PCR Mix (Cat#RR420A; Takara, Japan) in conjunction with the CFX Connect (Bio-Rad, USA) as previous study [44]. The 2^−ΔΔCT^ approach was utilized for data analysis, and each experiment was carried out with three separate sets of controls. The primer sequences used in this work were all synthesized by Sango Biotech (Shanghai, China), and they were all listed in the Table 1.

**Table 1:** Primer sequences.

**Gene name**	**Primer sequences (5′–3′)**
PDK2	Forward Primer: ATGAAAGAGATCAACCTGCTTCC
	Reverse Primer: GGCTCTGGACATACCAGCTC
ALDH5A1	Forward Primer: GACCTTGCCAGAATAATCACAGC
	Reverse Primer: GGGTGTGGATAATGTCTCCGT
CHDH	Forward Primer: TATCACCCTCCATTCAGCACA
	Reverse Primer: GAACCCACCTGTTTCCAGATG
GAPDH	Forward Primer: GGAGCGAGATCCCTCCAAAAT
	Reverse Primer: GGCTGTTGTCATACTTCTCATGG

### Histopathological and immunofluorescence

Tissue samples were fixed in a 4% paraformaldehyde fixation solution, embedded in paraffin, cut into 5-μm sections with a microtome, fully dewaxed, and hydrated. A hematoxylin-eosin staining solution was used to produce HE sections. Finally, we conducted the observation and evaluation using a microscope.

The immunofluorescence technique was conducted following the previously described protocol [45]. In summary, colon tissues were fixed in 4% paraformaldehyde at 4°C overnight, followed by immersion in 30% sucrose until they reached the bottom. Subsequently, the colons were embedded in optimal cutting temperature compound and frozen. Slices with a thickness of 10 μm were sequentially obtained at −20°C using a cryostat. These slices were then permeabilized in PBS containing 0.3% Triton X-100 for 30 minutes and blocked in PBS with 5% donkey serum at 37°C for 30 minutes.

Next, each section of the colon was subjected to overnight incubation at 4°C with the primary antibodies, namely ALDH5A1 polyclonal antibody (17319-1-AP), CHDH polyclonal antibody (17356-1-AP), PDK2 polyclonal antibody (15647-1-AP), and CD86 polyclonal antibody (13395-1-AP). Following this, the sections were washed and exposed to the corresponding secondary antibodies conjugated with fluorescence for a duration of 1 hour at 37°C. The nucleus was counterstained with 4,6-diamino-2-phenylindole (DAPI, Biosharp). The immunoreactivity density of ALDH5A1, CHDH, PDK2, and CD86 was quantified using ImageJ software (National Institutes of Health, MD, USA).

### Protein extraction and western blotting

Cells and tissues were subjected to protein extraction. Subsequently, the extracted samples were subjected to electrophoresis using a 10% SDS-PAGE gel and transferred onto a polyvinylidene fluoride membrane. Following this, the membranes were blocked with protein-free rapid blocking buffer (EpiZyme, Shanghai, China) for 0.5 hours. The membranes were then incubated overnight at 4°C with the following antibodies: ALDH5A1 polyclonal antibody (17319-1-AP), CHDH polyclonal antibody (17356-1-AP), PDK2 polyclonal antibody (15647-1-AP), and GAPDH (60004-1-Ig) from ProteinTech group (Wuhan, China).

### Data and statistical analysis

All statistical analyses were performed using R version 4.0.1 and GraphPad Prism 8.0 statistical software (La Jolla, CA, USA). The data in this study are presented as mean ± SD. Comparisons between two groups were conducted using Student’s *t*-test, Mann-Whitney *U* test, or Dunnett’s *t*-test, as appropriate. Differences between three or more groups were assessed using analysis of variance, and multiple comparisons between different groups were carried out using ordinary 1- or 2-way analysis of variance with a Sidak test. Statistical significance was defined as *P* < 0.05.

### Availability of data and material (ADM)

The Supplementary Material for this article can be found online at this study, further inquiries can be directed to the corresponding authors. The datasets analyzed during the current study are available online from GSE179285, GSE107499, GSE92415 and GSE48634 dataset.

## Supplementary Materials

Supplementary Figure 1
