# Supplementary material for: Mitochondrial-related genes PDK2, CHDH, and ALDH5A1 served as a diagnostic signature and correlated with immune cell infiltration in ulcerative colitis
Source: Aging (Albany NY). 2024 Feb 19;16(4):3803–22. doi: 10.18632/aging.205561 (PMC10929806; doi:10.18632/aging.205561)
Supplement: Supplementary Figure 1 [file aging-16-205561-s001.pdf]

SUPPLEMENTARY FIGURE

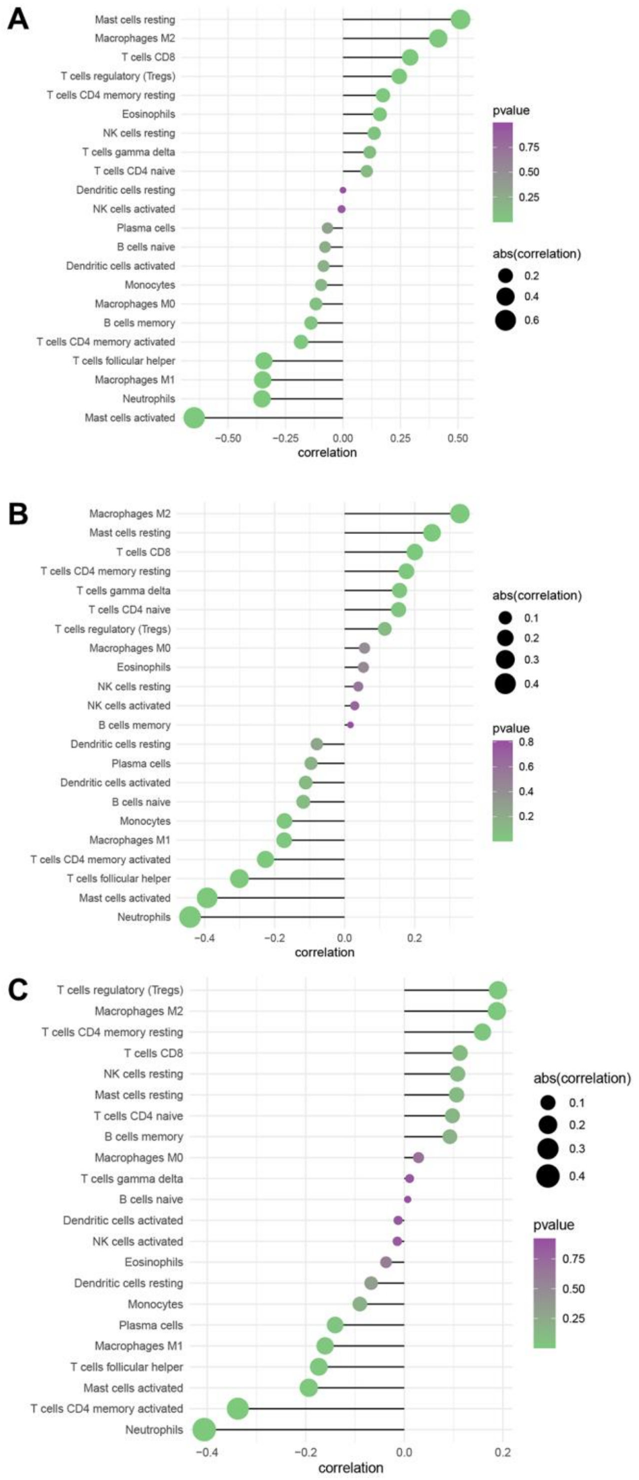

Supplementary Figure 1. The relationship between infiltration levels of immune cells and expression of (**A**) PDK2, (**B**) CHDH, and (**C**) ALDH5A1 by xCELL algorithm.
